# Forging just ecologies: 25 years of urban long-term ecological research collaboration

**DOI:** 10.1007/s13280-023-01938-w

**Published:** 2024-04-20

**Authors:** Morgan Grove, Steward Pickett, Christopher G. Boone, Geoffrey L. Buckley, Pippin Anderson, Fushcia-Ann Hoover, Ariel E. Lugo, Elvia Meléndez-Ackerman, Tischa A. Muñoz-Erickson, Harini Nagendra, L. Kidany Selles

**Affiliations:** 1 USDA Forest Service, 5523 Research Park Drive, Suite 350, Baltimore, MD 21218 USA; 2 Cary Institute of Ecosystem Studies, Box AB, Millbrook, NY 12545 USA; 3 School of Sustainability, Arizona State University, PO Box 877904, Tempe, AZ 85287-7904 USA; 4 Honors Tutorial College, Ohio University, 1 Ohio University Drive, Athens, OH 45701-2979 USA; 5 Department of Environmental and Geographical Science, University of Cape Town, Rondebosch, Private Bag x3, Cape Town, 7701 South Africa; 6 University of North Carolina, Charlotte, 9201 University City Blvd, Charlotte, NC 28223 USA; 7 USDA Forest Service International Institute of Tropical Forestry, San Juan, PR USA; 8 Department of Environmental Sciences, The University of Puerto Rico, Rio Piedras, 17 Ave Universidad STE 1701, San Juan, PR 00925-2537 USA; 9 International Institute of Tropical Forestry, USDA Forest Service, 1201 Calle Ceiba, Jardín Botánico Sur, Río Piedras, PR 00926 USA; 10 Centre for Climate Change and Sustainability, Azim Premji University, Burugunte Village, Bikkanahalli Main Road, Sarjapura, Bengaluru, 562125 India; 11 University of Puerto Rico, Rio Piedras, Facundo Bueso Building (FB-003) 17 Ave. Universidad STE 1701, San Juan, PR 00925-2537 USA

**Keywords:** Baltimore, Ecology, Environmental justice, Equity, Racism, Urban

## Abstract

We ask how environmental justice and urban ecology have influenced one another over the past 25 years in the context of the US Long-Term Ecological Research (LTER) program and Baltimore Ecosystem Study (BES) project. BES began after environmental justice emerged through activism and scholarship in the 1980s but spans a period of increasing awareness among ecologists and environmental practitioners. The work in Baltimore provides a detailed example of how ecological research has been affected by a growing understanding of environmental justice. The shift shows how unjust environmental outcomes emerge and are reinforced over time by systemic discrimination and exclusion. We do not comprehensively review the literature on environmental justice in urban ecology but do present four brief cases from the Caribbean, Africa, and Asia, to illustrate the global relevance of the topic. The example cases demonstrate the necessity for continuous engagement with communities in addressing environmental problem solving.

## Introduction

Urban ecology has gained prominence as a field of research and practice as the planet’s human population became majority urban. By 2006, half of the world’s human population lived in cities. By the middle of this century, more than two-thirds of people will call urban places home (United Nations Department of Economic and Social Affairs Population Division 2019). Cities, and urban places more broadly, have ecologies, and as habitats to most of the world’s human population, it is critical that ecologists contribute to understanding how these human-dominated and designed ecosystems function. This approach goes beyond monitoring and understanding how the clearly biophysical systems or “green” and “blue” patches are structured and function in urban areas. Such a biophysical approach has been termed the ecology *in* cities (Childers et al. 2015; Frantzeskaki et al. 2024). An expanded mandate for urban ecology is to monitor and comprehend the complex interactions between human and biophysical components of integrated urban systems, that is, the ecology *of* cities (Pickett et al. 1997; Grimm and Redman 2004).

An important program that fundamentally changed the field of urban ecology is the Long-Term Ecological Research (LTER) program of the US National Science Foundation (NSF; Kingsland 2005; Willig and Walker 2016). Although we illustrate the co-evolution of urban ecology and environmental justice using a US example, efforts in long-term ecological research are international (Vanderbilt and Gaiser 2017). For example, long-term urban studies exist in Beijing (Beijing Urban Ecosystem Research Station 2023), Strasbourg, France (l’Institut Ecologie et Environnement [INEE] du CNRS 2023), and Valdivia, Chile (Vera 2015), which show the global relevance of the approach. We complement a case study in Baltimore with four additional examples representing a broad range of urban places. The LTER program, which supported our core Baltimore case study for more than 25 years, was established in 1980 to ensure that long-lasting, episodic, or slow processes were included in the funding portfolio of NSF. Prior to that time, ecological research grants were typically one to three years in duration. This situation left many important ecological processes, such as natural disturbance, community and ecosystem succession, accumulated legacies, or indirect effects poorly documented and understood (Likens 1989; Lindenmayer et al. 2015). The LTER network began with sites focused on what might be called the native biomes of the US and its territories, defined by biological, climatic, and landform features. Examples included northern and southern deciduous forests, temperate rainforest, alpine tundra, arctic tundra, warm desert, tall grass prairie, short grass steppe, riverine, and wetlands.^1^ An agricultural site was established in 1982, soon after the start of the program. The history of the network has been detailed elsewhere using historical and sociological scholarship (Waide and Kingsland 2021).

After the first decade of LTER network activities, a blue-ribbon committee was convened in 1993 to assess progress and envision future development (Risser and Lubchenco 2003). That committee recommended that the LTER network be extended to include urban sites. This recommendation became reality in 1997 with a call for “up to two” urban LTER sites (Grove and Pickett 2021). This seemingly cautious request for proposals (RFP) resulted in the establishment of the Central Arizona Phoenix (CAP; Collins et al. 2000; Grimm and Redman 2004; Childers et al. 2019), and the Baltimore Ecosystem Study (BES) LTER projects. The RFP required three new things of urban sites (Grove and Pickett 2021):

In addition to the traditional LTER core areas, an Urban LTER will: 1) Examine the human impact on land use and land-cover change in urban systems and relate these effects to ecosystem dynamics; 2) Monitor the effects of human-environmental interactions in urban systems, develop appropriate tools (such as GIS) for data collection and analysis of socio-economic and ecosystem data, and develop integrated approaches to linking human and natural systems in an urban ecosystem environment; and 3) Integrate research with local K-12 educational systems.

The “core areas” are standard ecosystem measurements, developed to serve the natural biome thinking. The core areas are (1) pattern and control of primary production, (2) spatial and temporal distribution of populations representing ecosystem trophic structure, (3) pattern and control of organic matter accumulation, (4) patterns of inorganic inputs and movements of nutrients through soils, groundwater, and surface waters, and (5) patterns and frequency of disturbance. When the LTER network was being established, the fifth topic, disturbance, was just becoming non-controversial and widely recognized as important in the discipline. Still problematic in the late 1990s, however, was ecology’s wariness of incorporating humans into its agenda of feedbacks and adaptations (Kingsland 2005; Waide and Kingsland 2021). The two urban LTERs established in 1997 took the radical step of addressing humans—their institutions, technologies, cultures, and lifeways—as fully fledged factors within urban ecosystems (Machlis et al. 1997).

## Including humans and their effects in ecosystem research

For the LTER Network, the inclusion of humans in ecosystem studies was a momentous event that rippled throughout the discipline. It required incorporating new ways of thinking and data collection into ecology (Collins et al. 2000; Cadenasso et al. 2006). Few ecologists had thought deeply about integrating humans and their artifacts into empirical work. There were precedents on which BES and CAP could build, however. For example, a budgetary “metabolic” approach to ecosystem flows and their effects on human well-being had been applied to Hong Kong (Boyden 1976). An integrated approach that spanned ecosystem, community, population, and landscape approaches had been explored by a multidisciplinary Cary Conference entitled, “Humans as Components of Ecosystems” (McDonnell and Pickett 1993), although the scope was not specifically urban.

Another fundamental concept that shaped the Baltimore Ecosystem Study was the human ecosystem model (Machlis et al. 1997; Burch et al. 2017). This conceptual model stated that the human ecosystem comprised a social system and a resource system, connected by material, energetic, and informational flows. The human ecosystem model helped alert biological ecologists to the importance of social processes and features well beyond simple human demography and economy, which had been the usual currency of interdisciplinary connection. Another concept, the “total human ecosystem,” identified multiple dimensions of integration, ranging from culture to technology to biology, but it had not been widely adopted in ecology (Naveh 2000) (see also Box 4 in Pickett et al. 2024). A sabbatical stay by Naveh at Rutgers University in 1984 exposed Pickett to this concept.

**Fig. 1:**
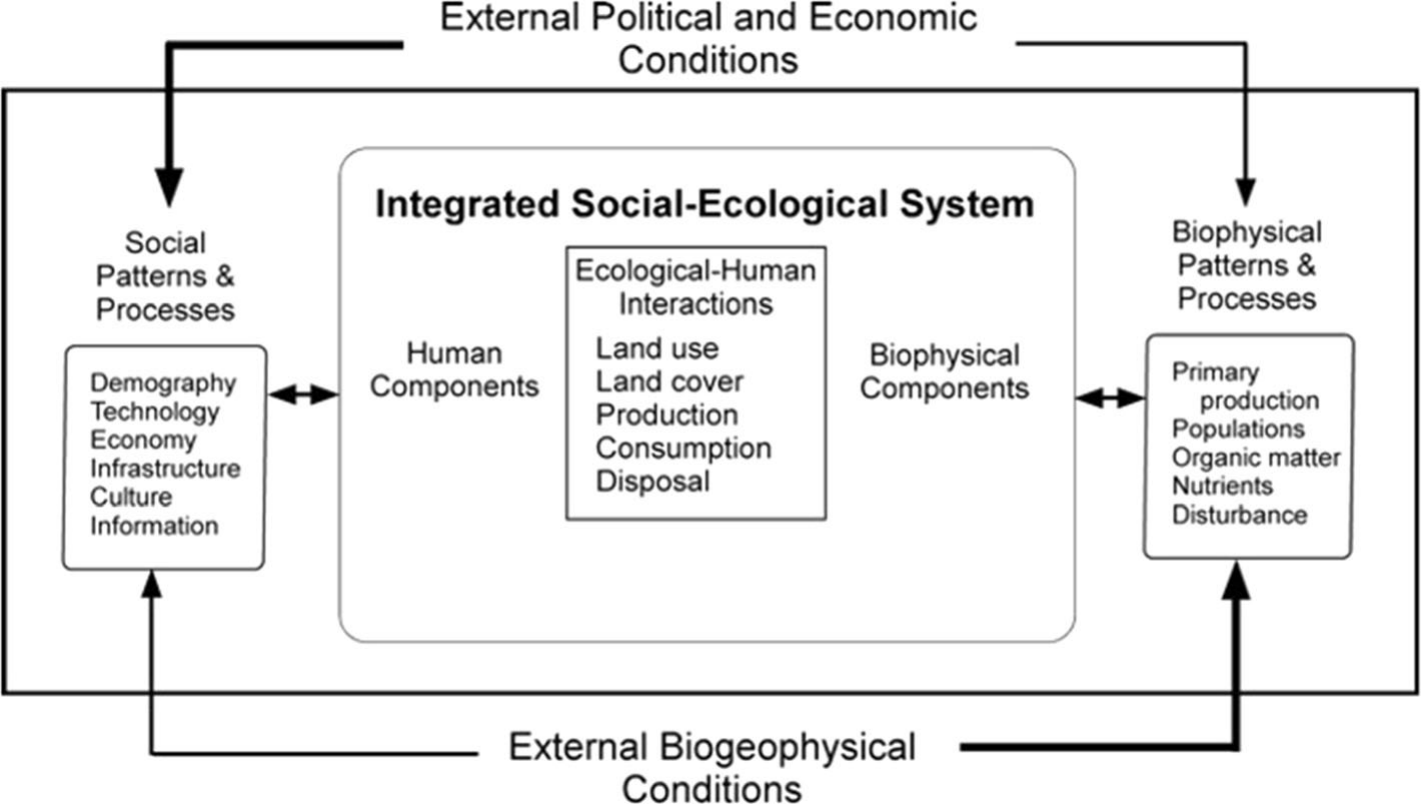
An early social–ecological framework in the LTER Program. An integrated social–ecological framework identifies external conditions that may be political, economic, or biogeophysical conditions; and social and ecological patterns and processes that interact with an integrated social–ecological system. Importantly, components within the integrated social–ecological system are considered both human and ecological simultaneously

**Fig. 2:**
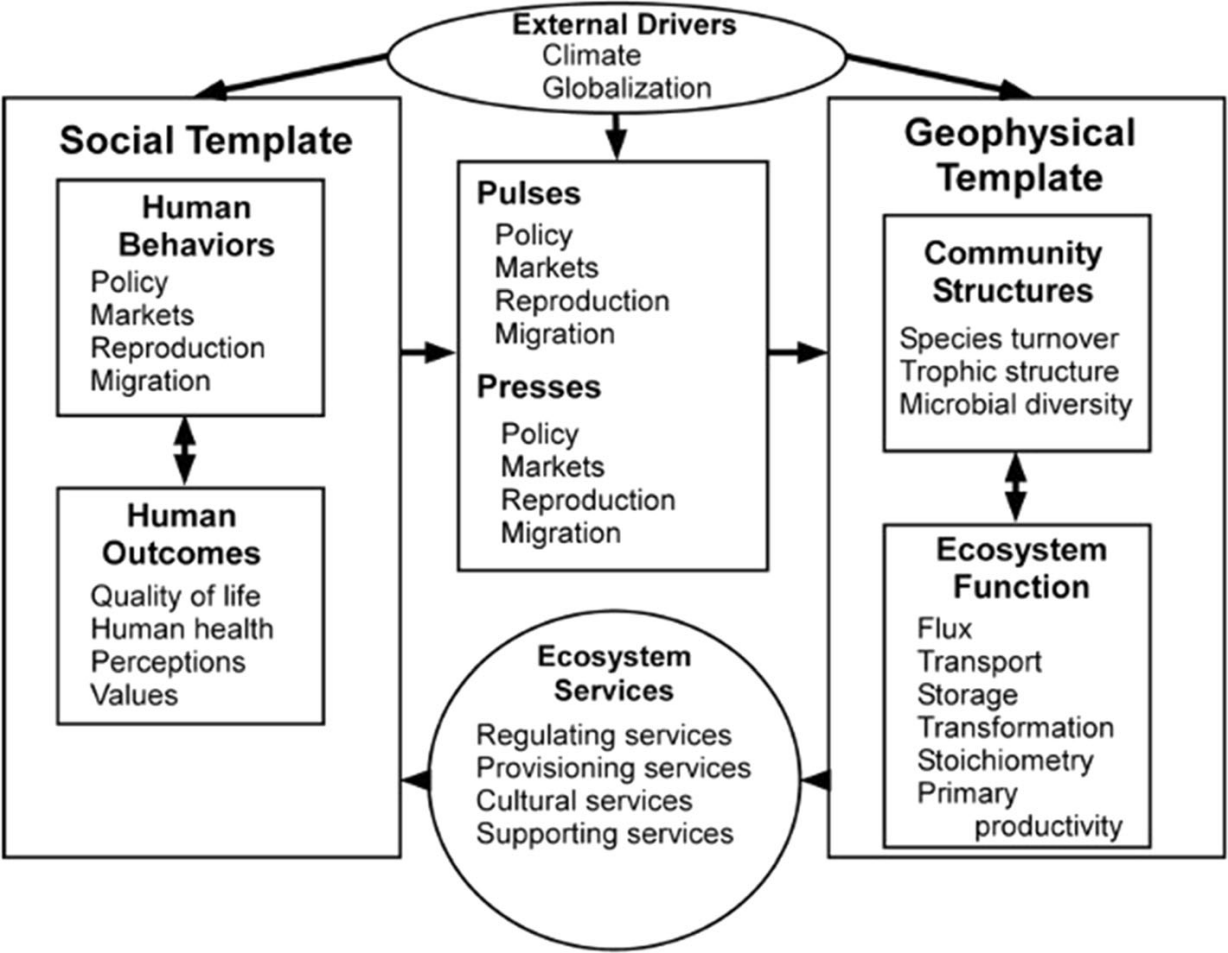
Intended to be an update to the Redman et al. (2004) integrated framework, the Integrative Science for Society and Environment (ISSE) framework was devised by an interdisciplinary group of social and biophysical researchers and policy makers. The framework was intended to give equal weight to social and biophysical structures and processes, to clearly identify services of relevance to humans, to show that biophysical and social processes and structures are reciprocally linked, that changes in the drivers of the system can occur as short term, or as chronic. (From Collins et al. 2011)

Once the first two urban LTER projects were established, interest burgeoned in suturing social and biophysical (ecological) approaches together. Indeed, the LTER Network itself sponsored workshops to explore social–ecological integration. Two products stand out: one by Redman et al. (2004; see Fig. 1) placed the social and biophysical components on equal footing and connected them through land use, land cover, production, consumption, and disposal; second, by Scott Collins (Collins et al. 2011) who had been instrumental in alerting NSF to the significance of urban ecosystems, presented interaction between the social and the biophysical realms as a cycle of interaction and consequence that operated through time (Fig. 2). LTER Network workshops exploring the cross-site comparability of disturbance as one of the five core areas of LTER research have been valuable in integrating social processes with biophysical ones as well (Peters et al. 2011; Grimm et al. 2017). (Box 1).

**Box 1:** International Influences and Interactions in BES

European and Israeli influences in landscape ecology were important to the initial conceptualization of the Baltimore Ecosystem Study (BES). In particular, Zev Naveh, Paul Opdam, and Isaak Zonneveld were significant to BES through their publications (Naveh and Lieberman 1989; Zonneveld 1989, 1990; Vos and Opdam 1993) and personal interactions with Naveh and Opdam. A sabbatical by Naveh at Rutgers University in 1984 exposed Pickett to the Total Human Ecosystem (THE) concept as well as an opportunity to meet Opdam during a visit to the United States at that time. BES employed the concept of Patch Dynamics (Pickett and White 1985) with landscape ecology to engage urban social ecological systems as spatially heterogenous, multi-scaled, and interdisciplinary systems. Importantly, BES embraced the landscape ecology idea that its urban ecology work should have practical applications as an actionable science to improve societal and ecological conditions.
BES was also influenced by international programs in rural, community development, particularly community forestry (RRA 1987; Lee et al. 1990; Cernea 1991). In addition to the idea of an actionable science, the emerging field of community forestry emphasized the importance of stakeholder engagement through Participatory Action Research and the differential power relations that could be present due to class, caste, and gender (Cernea 1991).
Soon after BES was established in 1998, it engaged with its sibling urban LTER, the Central Arizona-Phoenix (CAP) LTER and the Social, Behavioral, and Economic Directorate of the U.S. National Science Foundation (NSF) to explore the prospects for social sciences and humanities in the LTER Network. Sander van der Leeuw participated in a joint workshop, representing the French long term research program, Les Zone Ateliers (ZAs). From this workshop, Redman et al. (2004) identified a set of core social science research areas and a conceptual model for social-ecological research.
The interactions between BES and CAP and the French ZA projects grew over time. In the case of BES, scientists learned from these interactions to focus on the long term history of the city and the role of the state (government). In particular, it was essential to recognize that government and governance were not background to social-ecological systems; they were active drivers in social-ecological structures and functions. During this time, Boone (BES) collaborated with Sabine Barles and the Piren-Seine ZA to understand the comparative, social-ecological histories of stormwater management in the two urban regions (Boone 2003). Boone continued to work internationally, participating in a workshop to elucidate the emerging directions for the European Long-Term Social-Ecological Research (LTSER) network (Haberl et al. 2006).
Interactions among LTSER programs have continued internationally, although the U.S. funding agency, NSF, has refused to consider renaming the LTER network to indicate a social component. Singh et al. (2013) landmark publication, “Long term socio-ecological research: studies in society-nature interactions across spatial and temporal scales” highlighted the evolution and convergence of LTSER research internationally, particularly the importance of and methods for stakeholder engagement (Stringer et al. 2006; Reed 2008) and transdisciplinary science (Pohl and Hadorn 2008; Brandt et al. 2013). LTSERs, BES, and CAP are increasingly addressing the societal concerns for urban sustainability and resilience (Holzer et al. 2018; Holzer and Orenstein 2023).

Yet, these early efforts at integrating the social and the bioecological left out important things, perhaps due in part to some social science approaches –especially in economics–of simplifying models to a level where all persons are assumed to be equally well informed and have equal access to financial capital and to the levers of decision-making power. Similarly, researchers sometimes assumed that the mean condition of a geographic unit was an adequate representation of the population there. Social science often presented explanations and hypotheses in terms of “the usual suspects:” income, education or class, and race. While this is a start in acknowledging the heterogeneity of the social components of urban ecosystems, the usual suspects might erroneously be taken as fixed, essentialist characteristics of people or places (National Academies of Sciences 2023).

## The interaction of environmental justice and urban ecology

The BES and CAP urban LTERs began by initially following NSF’s requirements for incorporating humans into their work in rather neutral ways, but the reality of the racially, economically, and culturally segregated places the researchers worked in drove them to incorporate the causes, needs, and mechanisms of environmental justice in their work (Childers et al. 2014; Cadenasso and Pickett 2018). Insights on the social nature of racialized hierarchies, the pervasiveness of their ecological outcomes, and their persistence through time (Grove et al. 2018) were not part of the thinking evident in the non-urban LTER sites in the 1990s. Indeed, it has taken time for the urban LTERs to build empirical foundations, transdisciplinary connections, and deep engagement with affected communities or populations to fully shift to embrace environmental justice. Just as Gary Machlis, lead author of the “human ecosystem” framework, has said that no social model that neglects greed can be complete, so we have discovered that no urban social–ecological model that neglects racialized, class-based, and other criteria of oppression and exclusion is complete (Box 2).

**Box 2:** Hierarchies of social difference as the root of environmental injustice

Rather than being a biological given, contemporary research reveals race to be a socially and politically constructed condition. This does not deny that people have inherited characteristics, but that the hierarchy exploiting those characteristics, such as skin color or other aspects of physiognomy, is entirely a social product. In the United States, the hierarchy is derived from the ideology of white supremacy (Pulido 2015; Bratman and DeLince 2022). In other countries, light-complexioned people are afforded higher social status, regardless of any racial classification. Such “colorism” exists in Asian and South American contexts, for example. Hence, speaking of people who are *racialized* by social, cultural, and legal processes is a more appropriate conceptualization than assuming race to be a biological category (Yudell 2014). Similarly, people’s economic or class status may be socially constructed and politically reinforced (Hackworth 2019). For example, educational “attainment” in minoritized districts can be constrained in part by school financing arrangements—e.g., local property taxes in the U.S., or requiring school fees to attend public schools in some African counties. Environmental stressors or contaminants are also recognized to influence educational attainment (Sampson and Winter 2016; Schwarz et al. 2016).	

BES and CAP began to shift their attention to include environmental justice due to direct observation of segregation along with the emerging scholarship of race and class at that time (Bolin et al. 2000, 2005; Boone 2002). For example, driving or walking transects across Baltimore immediately alerted us to racialized segregation, disinvestment, extraction of wealth by landlords and speculators, lack of employment opportunities, and sparse recreational green space in minoritized neighborhoods. The social scientists in the Baltimore Ecosystem Study, including geographers and historians, pursued research that exposed the origin of contemporary racialized exclusions. They discovered that even past exclusions have legacies that echo into the present, as we will detail later. Furthermore, working closely with community and municipal leaders revealed intense, ongoing community-based work to achieve social and environmental justice in many marginalized neighborhoods.

One of the benefits of exploring the human and biophysical interactions in the urban LTERs has been engagement with diverse stakeholders, including municipalities, community members, non-profit organizations, social and biophysical scientists, engineers, and humanists. These interactions have provided fertile ground for interdisciplinary and transdisciplinary forms of inquiry, blending of theories, methods, and epistemologies from diverse scholarly and practice fields, and participation in basic, applied, and normative research (Rademacher et al. 2023). In this paper, we argue that the fields and concerns of environmental justice and urban ecology have both benefited substantially from such blending. Among other advances, the interactions between environmental justice and urban ecology have forged a new ecology *with* cities, a transdisciplinary approach that takes into consideration the needs and desires of urban dwellers in order to develop strategies with stakeholders to design and implement just and sustainable futures. Urban ecology *with* cities embraces ethical and social justice perspectives, inspired by environmental justice and practice, as transformative ideas that lead to innovative science and desired community outcomes (Pickett et al. 2021).

Environmental justice has likewise benefitted from urban ecological approaches. Early work in environmental justice, for justifiable reasons, focused on the negative consequences of environmental harms on minoritized groups because of the risks to health and well-being they created (Bullard 1983; Bullard and Wright 1993). These environmental harms or burdens were primarily the products of industrial production, such as the release of toxics and pollutants into the air, or the mismanagement of wastes, such as landfills or water pollution. Urban ecology reminds us that urban ecosystems produce benefits, or services, that can improve human well-being. However, the heterogeneity of urban ecosystems along with spatialized race and class segregation in cities means that these ecosystem services are not experienced evenly (Kabisch and Haase 2014). Using an environmental justice lens, it is possible to examine the distribution of ecosystem services as environmental goods or amenities in relation to group characteristics of neighborhoods. Thus, the uneven distribution of tree canopy cover, green space, or good water and air quality as an environmental justice issue can be tied back to metrics of ecosystem services (Bargmann 2013; Pickett et al. 2013; Schwarz and Manceur 2015; Locke et al. 2021a, b).

The integration of urban ecology and environmental justice has strengthened our understanding of how cities are structured, who bears the burdens and enjoys the benefits of environmental bads and goods, and how ecosystem services could be redistributed to lessen environmental injustices. These types of inquiries focus on distributive justice, or the fairness of how benefits and burdens are spatially distributed among individuals or groups. Most environmental justice studies include distributive justice analyses, typically by examining the spatial distribution of environmental amenities and disamenities in relation to the population characteristics of neighborhoods. Since the landmark study by Robert Bullard (1983) of landfill siting in Houston in the late 1970s, the conclusion from the majority of distributive justice studies shows that racial and ethnic minoritized populations bear a disproportionate burden of environmental harms (Mohai and Saha 2007; Bullard et al. 2008).

These distributive justice findings have prompted researchers to ask how these patterns came to be and what allows them to persist. Robert Bullard recognized that the majority of landfills located in Black neighborhoods in Houston were the product of racism, which because of its spatial and environmental context, he modified as environmental racism. Building upon Bullard’s pioneering work, environmental justice studies have increasingly examined historical processes. Research that examines the mechanisms by which unjust distributional patterns come to be are labeled procedural justice. Many mechanisms, such as redlining, housing discrimination, zoning, infrastructure, and other tools that concentrated environmental harms and reduced environmental goods in minoritized neighborhoods long before the present-day, contribute to procedural injustice (Boone et al. 2009; Grove et al. 2018). Procedural justice studies have also underscored the importance of fair processes over time, regardless of distributional outcomes, as environmental justice concerns.

Related to procedural justice is the need to recognize and include all communities in environmental decision-making (Whyte 2011). Recognition justice demands that communities, particularly the most vulnerable, should be actively included in planning, decision-making, and implementation of public investments. In particular, recognition justice requires not only that all communities and groups are *present* in the processes, but that their voices are in fact *listened to* and their knowledge is *legitimized* (Nightingale 2017). The call for recognition justice stems from ethical considerations of doing the right thing, but also the expectation that recognition and inclusion will result in better outcomes. Since public investments in parks, tree canopy cover, stream restoration, or brownfield development will likely last decades, the needs and concerns of future generations also play a key role in determining just processes and outcomes. The ability to think about upstream and downstream consequences of local decisions has become another important aspect of environmental justice. Thinking about how local strategies will affect surrounding communities, from regional to global scales, is an environmental justice consideration that increasingly informs sustainability planning (Seto et al. 2012). The climate justice movement is an important example of how inter-generational justice and local-to-global justice are key criteria for assessing fairness (Boone and Klinsky 2016).

Urban ecology can also engage environmental justice by informing how interventions, such as the expansion of park space or tree cover to meet environmental justice needs, will have higher chances of success if they employ scientific principles for effective ecosystem structure and function, such as the use of appropriate tree species for specific site conditions (Warren et al. 2010; Pickett et al. 2013). How to create wider societal benefits by connecting greenspace planning to other ecosystem services, such as clean air and water, pollination, or cultural ecosystem services, are additional realms where urban ecology may intersect effectively with environmental justice. These intersections among environmental justice, urban ecology solutions, and decision-making are likely to increase as policies, plans, and management seek to address both historic inequities and adapt to future climate change (Elmqvist et al. 2021).

The general understanding of how rank hierarchies of oppression lead to injustice, and the relationship of ecology to environmental justice is operationalized in Baltimore, as the following section details.

## Surprising environmental justice results and long-term interactions

Twenty-five years of collaboration in BES among ecologists and a variety of social specialists have benefitted both groups intellectually, leading to better informed questions and an increasingly sophisticated understanding of the forces that produce landscapes of inequity over the long-term. Key to these interactions has been progress in developing our conceptual models of environmental justice and identifying the roles for ecologists to engage with environmental justice (Grove et al. 2013).

Our initial conceptualization of environmental justice focused on the distribution of disamenities and amenities in the contemporary spatial matrix of Baltimore. One of our early environmental justice studies found that toxic facilities were more likely to be located in White rather than Black neighborhoods (Boone 2002). This finding appeared to contradict environmental justice work in other locations, which found that toxic facilities were more often located in Black neighborhoods (Boone 2002). We also examined the distribution of tree canopy cover and found that it was positively correlated with Black neighborhoods (Grove et al. 2006; Troy et al. 2007). Again, this contradicted other environmental justice research, which had found a positive correlation of tree cover with White neighborhoods (Bullard 1990). Although we were initially puzzled by these findings, we were eventually able to better understand these contradictory results once we recognized and contextualized our findings with Baltimore’s long history of racialized housing practices, segregation, and exploitative real estate practices (Grove et al. 2018). We unpack these surprises below.

The inequitable distributions of environmental disamenities and amenities were intimately tied to the obvious pulses of racist policies; but the inexorable press of institutionalized racism was a revelation. More than a century of policies and practices that promote racialization and segregation have led to the accumulation of amenities in White and wealthy districts and the exclusion of investment in Black communities. Starting with passage of Baltimore’s segregation ordinance in 1910–1911, there was a spasm of racially restrictive activities in the city. These included neighborhood covenants, whereby house sales to racial or religious minorities were restricted; the comprehensive assessment and mapping of the risk of mortgage default in different neighborhoods of city by the Federal Government’s Home Owners Loan Corporation (HOLC) conducted in partnership with local “experts;” the blockbusting tactics of unscrupulous real estate agents that stoked white fear of residential integration and inordinately profiting from sales to Black residents; and the governmentally subsidized White migration to the suburbs. Together, these actions were major racialized drivers of privilege in one place and decline in another (Rothstein 2017; Brown 2021).

The distribution of toxic facilities and residence in Baltimore was the result of decades of racist housing practices that privileged Whites to live close to work, but kept Black Baltimoreans segregated and forced them to travel longer distance to their jobs in factories or the port. Similarly, the long-term history of Baltimore’s racist housing practices informed our understanding of the distribution of tree canopy cover. As White Baltimoreans left the city, through a combination of block busting in the city and the governmental subsidization of mortgage lending of suburban housing for Whites in the counties adjacent to Baltimore City, they left behind the trees and parks that had been part of their White privilege. Black Baltimoreans moved into these neighborhoods and “inherited” the environmental amenities of former White residents. Thus, Black Baltimoreans experienced these environmental amenities because of long-term processes of racist housing practices.

**Fig. 3:**
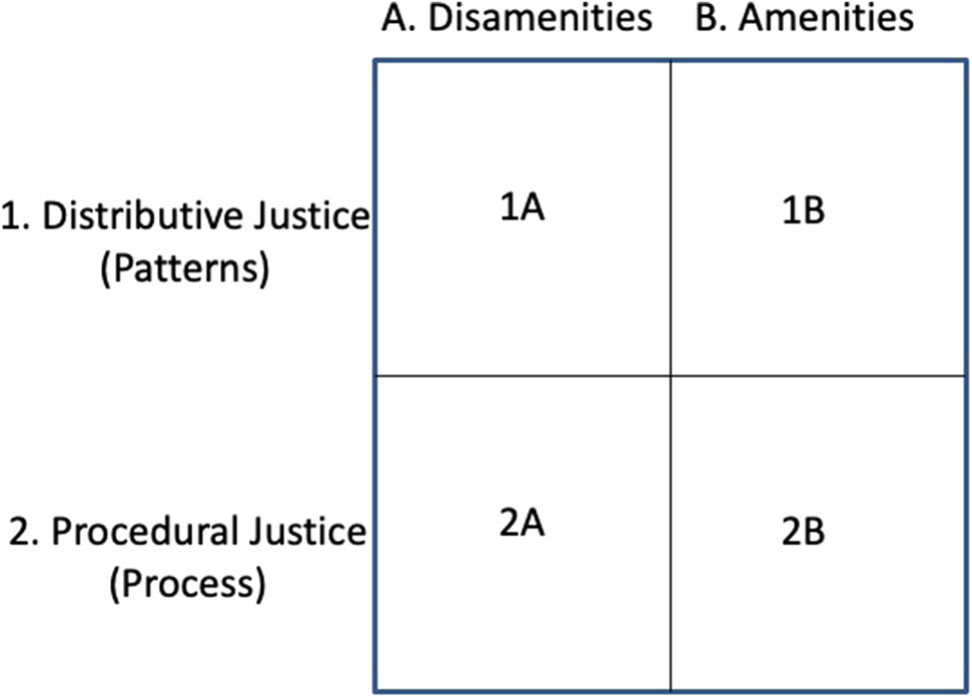
Environmental justice in BES was initially conceived of as the distribution of disamenities (1A) or amenities (2A); or the procedures for the inequitable distribution of disamenities (2A) or amenities (2B) in the contemporary spatial matrix. It is important to note that this framework did not consider either the history nor the interactions among the four categories. Examples of amenities include trees, playgrounds, parks, gardens, or greenways. Examples of disamenities are associated with pollution–air, water, odor, or noise–and include proximity to polluting industries, waste treatment plants or landfills, or major thoroughfares such as highways or railways

The inheritance of landscapes formerly established or tended on behalf of White residents and the role of historic racist housing practices alerted us to the need to extend the focus of environmental justice research from the contemporary spatial matrix of disamenities and amenities, to detailed, mechanistic examinations of procedural justice (Fig. 3). An important mechanism of injustice was the racially differential granting of environmentally damaging zoning variances in Black neighborhoods in Baltimore. Administrative and judicial documents shed light on how and why decisions permitted environmental zoning variances in Black neighborhoods but excluded from White neighborhoods (Lord and Norquist 2010). Further examples of historical records exposing the roots of injustice appear in the exclusion of Black golfers from city-owned courses. The formation of clubs in the Black community that brought pressure on recreational segregation in the city was crucial. Eventually, legal action led to a desegregation of all public golf courses in Baltimore City (Wells et al. 2012). Not all community agitation had the outcome desired by Black citizens, however. For example, there was a recognized dearth of playgrounds in Black neighborhoods in Baltimore in the 1930s. A much-hoped-for effort to establish playgrounds in Black neighborhoods failed when those funds were reallocated to purchase land for a large park adjacent to White neighborhoods (Korth and Buckley 2006).

**Fig. 4:**
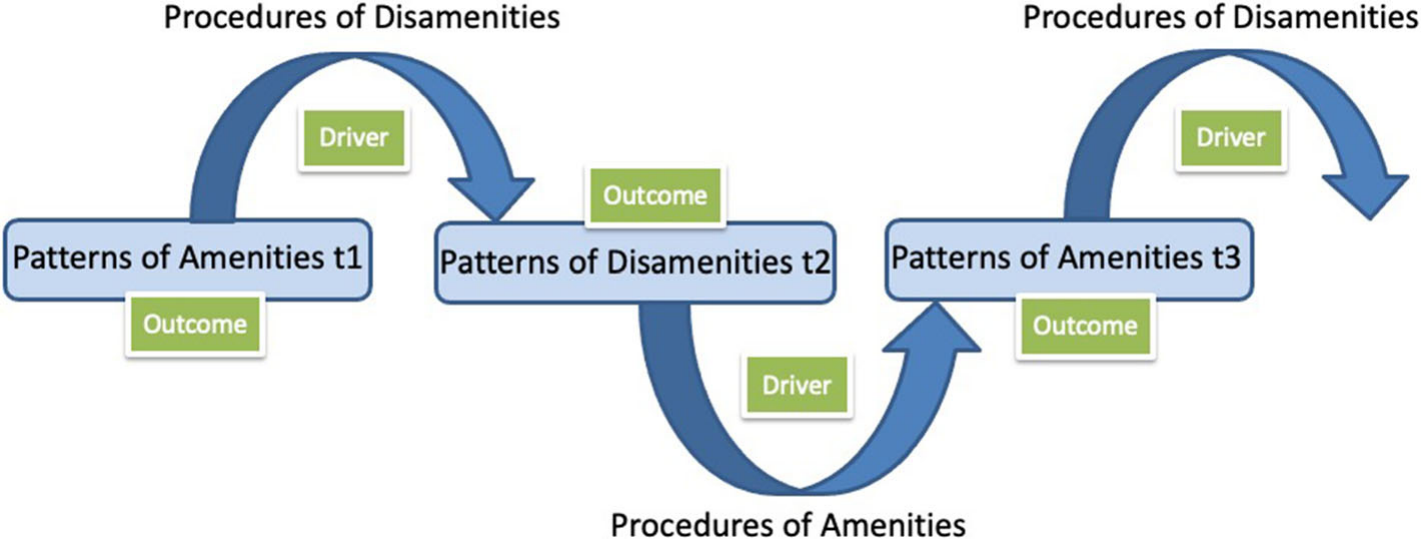
The dynamic heterogeneity framework can be used to explore interactions among distributive and procedural justice and amenities and disamenities. For example, patterns of amenities at t1 could affect the allocation of disamenities that produce patterns of disamenities at t2. Subsequently, patterns at t2 could affect the allocation of amenities for patterns of amenities at t3. The dynamic heterogeneity framework can be used to explore interactions among distributive and procedural justice and amenities and disamenities. For example, patterns of amenities—public parks, gardens, and street trees–at t1 could affect the procedural allocation of disamenities–zoning variances–that produce patterns of disamenities—polluting industries–at t2. Subsequently, patterns at t2 could affect the procedural allocation of amenities—decisions about the purchase of new park lands–for patterns of amenities at t3, which could lead in turn to procedural allocation of disamenities—recognition of environmental injustices and zoning reform (Boone et al. 2009; Lord and Norquist 2010). This sequence creates ecological heterogeneity in the distribution of vegetative and hydrologic structure and biodiversity, which affects ecosystem fluxes—quantity and quality—of air, water, and energy–that are fundamental to ecosystem services to the social system (Collins et al. 2011)

The linkages between the distribution and the procedures that allocated disamenities and amenities over time caused us to shift our conceptualization of environmental justice from a static spatial matrix view to what we called a “dynamic heterogeneity” view (Fig. 4), which connected distributional and procedural perspectives over time. In this case, environmental justice conditions in T_1_ were used for procedural decisions to create new environmental justice conditions in T_2_. Specifically, the conditions in T_1_ are part of the reasoning for allocating disamenities or amenities in T_2_.

Spatial zoning in the United States can play a role in this process over time. Zoning is an established governmental planning practice in the United States used to spatially prescribe and map which land use activities are allowed in particular parcels or zones. It was originally established to promote health and safety. For instance, zoning is used to separate polluting industries from residential areas instead of allowing the two uses to be intermingled. A zoning variance is a legal process that allows an activity in a parcel or lot even though it does not conform to the existing zoning rules for the larger area. For instance, zoning variances in Black neighborhoods were disproportionally approved because those Black neighborhoods were judged to be already degraded and the marginal cost would be low. In contrast, variances were disproportionally disapproved for White neighborhoods because those White neighborhoods were judged to be in good condition so the marginal cost would be high. In another example, the nationally known landscape architect, Frederick Law Olmsted, Jr., advised the City’s Recreation & Parks Board that playground investments in Black neighborhoods would be less valuable than park investments benefitting white neighborhoods (Korth and Buckley 2006).

Our use of dynamic heterogeneity to understand environmental justice provides an opening to understanding Baltimore’s racialized exclusions as part of a larger and more comprehensive complex adaptive system that includes social, economic, and ecological lags and legacies that can persist in urban ecological systems over time. For example, when we compare the 1937 HOLC maps of foreclosure risk by neighborhood in Baltimore with the 2017 distribution of canopy cover in those same neighborhoods, we find large differences. Neighborhoods which were exclusively white in 1937 and given high scores for low-risk mortgage lending—that is “green-lined”—had more than twice as much canopy cover in 2017 as contemporary neighborhoods that were predominantly Black and assessed as risky for mortgage lending, that is “red-lined” in 1937 (Locke et al. 2021a, b).

Over time, we have encountered many interconnected and reifying pathways that produce landscapes of privilege and oppression. We have applied our conceptualization of a dynamic heterogeneity perspective to a complex, systems view in order to be clear about the numerous social and ecological components of urban systems that reinforce institutionalized racism through multiple interactions. Thus, we conceive of environmental justice as part of an adaptive, complex, and resilient system of institutionalized racism. We use the terms “adaptive, complex, and resilient” to indicate that institutionalized racism consists of multiple, reinforcing positive and negative feedbacks (Grove et al. 2022; Pickett et al. 2024). Further, we have come to understand that institutionalized racism is a fundamental logic and driver of urban ecological systems and to ask how racism is so resilient in urban landscapes, using a systems perspective.

## How can ecologists better infuse ecology into environmental justice

The LTER program was established to study long-lasting, slow, or episodic processes. However, its focus on developing a long-term perspective led to our understanding of institutionalized racism as a complex, adaptive, and resilient system from the perspective of environmental justice. The terms “complex, adaptive, and resilient” combine with a systems perspective to serve as essential parts of an ecologist’s toolkit and framing. Thus, one of the fundamental roles for ecologists in environmental justice is to bring ecological thinking—such as systems thinking that requires identifying relevant system parts, as well as the interactions among the parts of a system, and interactions of a system of interest with other systems—to understand the dynamic feedbacks that make institutionalized racism so resilient. Understanding environmental racism as a system can help identify potential interventions to dismantle the racist system and create new alternatives.

A second role for ecologists is for them to help understand how institutionalized racism transforms ecological systems (Schell et al. 2020) from disease vectors such as mosquitos (LaDeau et al. 2013) to biodiversity (Burghardt et al. 2023), heat islands (Huang et al. 2011), and distribution of trees (Locke et al. 2021a, b; Anderson et al. 2023), to name a few. These efforts will be limited, however, unless we recognize that certain groups and their places are poorly understood because of racism or classism. This is the result of what Gadsden et al. (2022) call the “Landscape of Fear.” Specifically, this reflects the unwillingness of ecologists from privileged populations to work in places and with people with whom they do not feel comfortable or may even fear. Overcoming these blind spots will require new partnerships and practices that are fundamental to any transdisciplinary approaches for an urban ecology *with* cities. Finally, we suggest that ecologists are not passive observers of urban ecological systems. Ecologists have roles to play in society, using ecological thinking and ecological phenomena, to address environmental justice to remedy the past and prepare for the future.

An example of embedding ecologists in environmental justice work returns us to the concern with urban tree canopy in Baltimore. The city has adopted a policy of doubling its tree canopy by 2030. However, our research has shown that increasing urban tree canopy entails more than the identification of permeable space, selection of appropriate species, and placement of tree root balls in the ground. Installing street trees, especially, requires careful negotiation among stakeholders, including government officials, forestry professionals, non-profit groups, and residents. While Zhou et al. (2021) demonstrate that planting trees for cooling purposes in socially vulnerable neighborhoods is a social–ecological “win–win,” Battaglia et al. (2014) explain why residents in East Baltimore have long resisted such efforts, citing a variety of perceived disservices associated with trees, competing priorities, concerns about crime, and fear of gentrification.

Consequently, there is a roster of difficulties some communities may experience when confronted with the opportunity to green their neighborhoods with trees. For example, studies by Carmichael and McDonough (2019), Berland et al. (2020), Locke et al. (2021a, b), and Roman et al. (2021) underscore the importance of involving communities in decision-making processes. This is particularly relevant for non-profit organizations involved in urban greening but having few ties to the communities in which they work. As these and other authors show, failure to establish bonds of trust may result in residents exercising the one power they believe they possess–the right to refuse trees. In an era of diminishing municipal investment in green infrastructure, studies that explore the effectiveness of alliances between communities and non-profit organizations are needed to ensure the viability of long-term greening programs.

Parks are another green infrastructure requiring integration of social and ecological understanding. Our research shows that achieving a just distribution requires that we pay close attention to both distributive equity and procedural justice. That is, residents should not only enjoy the many and diverse benefits of park access, but they should also be involved in a meaningful way when it comes to making decisions that affect them. Our experience also highlights the value of adopting a historical approach to environmental justice investigations because only a long-term perspective allows us to recognize and understand the patterns we observe today. But these alone are not enough to truly ensure equity. The emergence of interactional justice–which focuses on how marginalized groups experience public spaces such as parks–coupled with a perspective that views parks as spaces of care, is an avenue of inquiry that will likely gain traction in future (Rigolon et al. 2018; Bonds and Holifield 2022).

Environmental justice remedies and plans for climate change adaptation will need to work to dismantle institutionalized racism as a resilient system as well as to build new and just ones. Urban ecologists have a role to inform, monitor, evaluate, and adapt policy, programs, and management aimed to address social and environmental equity. Programs such as the Federally supported “urban renewal” by demolition and replacement of old established neighborhoods in the US were ostensibly intended to improve soundness of housing, and to modernize urban infrastructure, but in many instances actually discriminated against impoverished people or people of color (Connolly 2014; Lieb 2018; Cebul 2020). Ecologists were absent from policies and programs to address equity during the US Federal Urban Renewal programs of the 1960s and 1970s. It is not clear whether ecologists are substantially involved today on a widespread basis at a local level for current policies and programs to advance urban sustainability and adaptation to climate change.

For ecologists to be substantially involved in planning and urban social–ecological revitalization, it is crucial that they help illuminate institutionalized racism as a complex, adaptive, and resilient system. This will require teams with a plurality of disciplines, perspectives, and skills to comprehensively identify and understand the diverse drivers and feedbacks in the system. Building teams of sufficient scope will be difficult and require long-term commitments.

Finally, the long-term perspective in BES helps us to appreciate that the fight is not new. Minoritized and marginalized communities have long engaged in actions to overcome racism (Dyson 2020; Gates and Louis 2020). As government officials, community leaders, and the allies of oppressed people and places take up the task of addressing environmental justice issues in our cities, it is imperative they deliver messages of hope that normalize participation in nature-based activities by minoritized populations. Known as “transgressions” because they offer a counterpoint to the dominant societal narrative and its associated historical trauma, examples of these stories are increasingly found in the literature (Algeo 2013; Theriault and Mowatt 2020; Dietsch et al. 2021; Dickerman and Buckley 2022).

An example of activities aimed at emancipation from segregationist systems is the struggle in Baltimore toward integrating public recreational facilities. Founded in 1938 at Carroll Park golf course, the Pitch & Putt Golf Club of Baltimore is one of the oldest African American women’s golf clubs in the US. Their efforts were instrumental in acknowledging the Black struggle to overcome the barriers of segregation of the city’s golf courses. This effort has now become part of the positive narrative of justice in Baltimore. In early 2022, after months of research, fundraising, and planning, its members unveiled a monument dedicated to the Black golfers, both male and female, who fought to gain access to the city’s public courses. According to a *Baltimore Sun* story dated on February 1, 2022: “Although Carroll Park was the only course open to Black golfers in the 1930s, 1940s, and 1950s, the absence of other options spurred legal action and protests that finally forced Baltimore officials to make its golf courses, baseball fields, swimming pools, and other public recreational facilities available to all people of color.” Ensuring that these stories are told and celebrating the history and achievements of people of color in these spaces is a critical first step in the process of redressing decades of environmental injustice and working for the future.

## Environmental justice research and action is a global concern

To this point, we have told the story of urban ecology’s shift toward including environmental justice in all its forms, from the perspective of long-term research in Baltimore. However, the kinds of segregation, marginalization, and exclusion from urban decision-making that have characterized Baltimore’s history of environmental justice also appear in different forms around the world (Bashi and Hughes 1997; Nightingale 2012, 2017). In this section, we present brief summaries of environmental justice and activism from the international realm. The “voice” of each contribution is retained for the sake of highlighting the local concerns and narrative power of each case. The cases are from a tropical city on the Caribbean Island of Puerto Rico, the City of Cape Town, South Africa, and Bangalore in India.

### Epistemic justice for the future of the Río Piedras watershed, San Juan, Puerto Rico

The Río Piedras River has played a crucial role in the development of the city of San Juan. As the only river in this tropical coastal city of San Juan, the Spanish constructed an aqueduct in the late 1800s that conveyed water from the river with a low-water dam and valve house to supply potable water through gravity to those living in what is now referred to as Old San Juan and surrounding farmlands (Sepúlveda Rivera 2016). Over time, the river has undergone significant changes due to development, wetland filling, and stream channel burial. For most of the city’s population and institutions, the river has gone unnoticed throughout the urban landscape. This changed when the island was battered by hurricanes Irma and María in 2017 and a project initially proposed in the 1980s to channelize the Río Piedras resurfaced as part of the federal government’s billion-dollar investment in disaster recovery, reconstruction, and mitigation in Puerto Rico. Local scientists and professionals have opposed the project, expressing concerns that channelization is an outdated solution to the city’s current social, ecological, and infrastructural conditions, as well as its climate-changed future. Furthermore, many residents living in neighborhoods along the river have criticized the planning process for ignoring their concerns, knowledge, and lived experiences with flooding. The Río Piedras has become the center of discussions about the city’s future and the development pathways that will determine options for climate adaptation and resilience.

The fate of the Río Piedras brings to the fore the many environmental justice challenges involved in disaster recovery and resilience-building efforts. Specifically, whose knowledge counts? As urban social–ecological systems scientists we’ve been studying the river, its watershed, and its human and non-human communities for over a decade (Muñoz-Erickson et al. 2014). We’ve also studied the different knowledge systems that inform flood management in San Juan. These knowledge systems encompass the practices, social networks, sources of information, and methods that people and organizations use to understand and make sense of their urban environment and flood experiences (Ramsey et al. 2019). This includes the knowledge systems that residents in communities along the Río Piedras use to understand and adapt to flooding. We have learned that, compared to the formal knowledge systems of local and federal agencies that manage coastal, riverine, and urban flooding separately, residents’ knowledge systems view flooding in an integrated way having a broader repertoire of solutions to adapt to flooding.

In recent years we have presented our findings to city managers and decision-makers in the expectation that this knowledge can help fill in gaps and avoid flood mitigation actions that may lead to maladaptation, irreversible ecological losses, and environmental injustices. We have realized that it is not enough to reveal the diversity of knowledge systems. It is also necessary to ensure epistemic justice as a principle that recognizes and includes these diverse perspectives and knowledge systems into planning and decision-making processes.

**Fig. 5:**
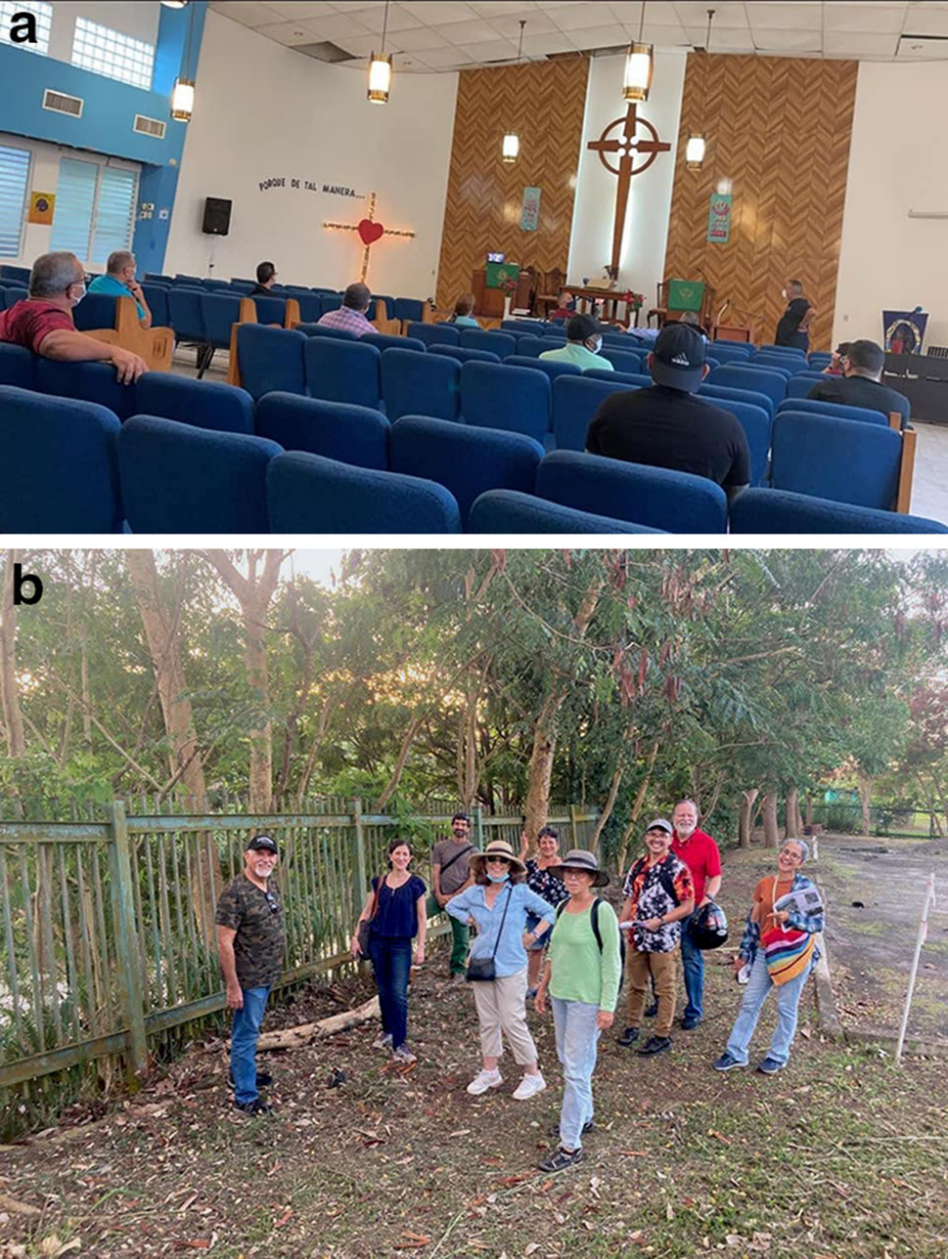
Examples of coproduction activities and spaces where scientists, residents, and community activists meet to exchange knowledge and experiences about the Río Piedras, including community organization meetings at the local church (left image) and field visits between upstream and downstream communities (right image) (Photo credit: Tischa Munoz-Erickson)

**Fig. 6:**
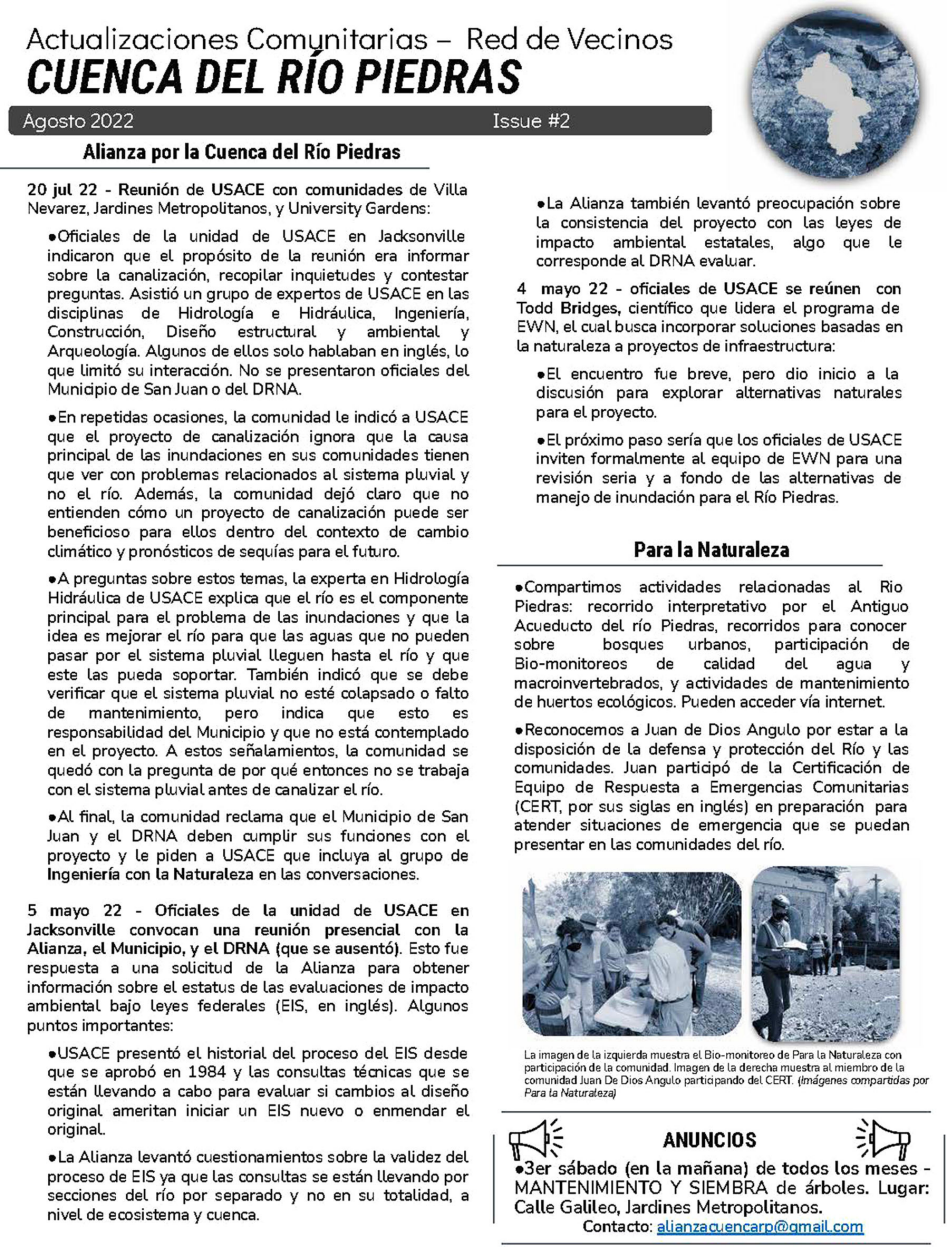
Image of one of the volumes of the bulletin that the *Alianza por la Cuenca del Río Piedras* coproduced with community leaders as a platform for information sharing and network building among upstream and downstream river communities

We are collaborating with the *Alianza por la Cuenca del Río Piedras* (Río Piedras Watershed Alliance; *Alianza*), a coalition of government representatives, non-governmental practitioners, planners and engineers, and community activists in a broader effort to connect and include these diverse knowledge systems in discussions about the channelization project. The coproduction process involves spaces and practices that center on building relationships and trust, and that promote knowledge sharing, social learning, and creative collaborations. These spaces and interventions have taken place in local churches, in field visits between upstream and downstream river communities, and primary school classrooms, for example (Fig. 5). We have realized the privilege role that our scientific knowledge systems have traditionally had and looked for ways to amplify other kinds of knowledge, such as developing a community bulletin for the different communities and interest groups to share their knowledge and actions related to the Río Piedras (Fig. 6). While our approach has added value to the interactions of communities with their government agencies, the challenge remains in finding room within entrenched government procedures for the novel ideas that emerge from a broader discussion of issues. Nevertheless, the coproduction process has transformed our role from scientists that study the urban watershed to being part of a community of practice where all members are considered knowledge producers and users looking out for the Río Piedras and the role it will play in San Juan’s future.

### Environmental justice transformations of urban ecology: Thoughts from the City of Cape Town

In any South African city an interrogation of environmental justice and urban ecology will always point to the persistent and damaging role of the apartheid urban form. While informed by longer colonial histories, and then entrenched under the apartheid regime, South African cities were set up to keep black people on the margins and in living conditions that signaled and reaffirmed a racially informed subclass. Certainly, this is the case in Cape Town, perceived as one of South Africa’s most segregated cities (Turok et al. 2021). Margins here are not only the geographic edges of the city, but also the most environmentally exposed areas, with for example a shallow water table that rises up in the winter and exposed mobile sand dunes that give rise to large volumes of atmospheric particulate matter in the high winds of summer.

To understand the role of spatial urban form in Cape Town and quantify what this means for associated urban ecologies, we sampled plants within one original biophysical template, across a socio-economic gradient that transitioned from wealthier, predominantly white neighborhoods to poorer, predominantly black neighborhoods (Anderson et al. 2020). What we found was a significant ecological gradient of reduced plant species and trait diversity across our gradient. Furthermore, wealthier communities benefitted from more private green space and more public green space while poorer communities had limited green space on all fronts. It is well known that plant communities with limited diversity are less resilient and, if exposed to environmental extremes as anticipated with global change, would lose species, and the associated ecosystem services, faster than a species-rich community. These species-poor plant communities mirror historical apartheid planning, in relation to a city form that is resistant to change (Turok et al. 2021). Based on how biodiversity, functionality, and associated ecosystem services and ecosystem stability are linked, this study shows how significant environmental injustices manifest and persist in the City of Cape Town.

**Fig. 7:**
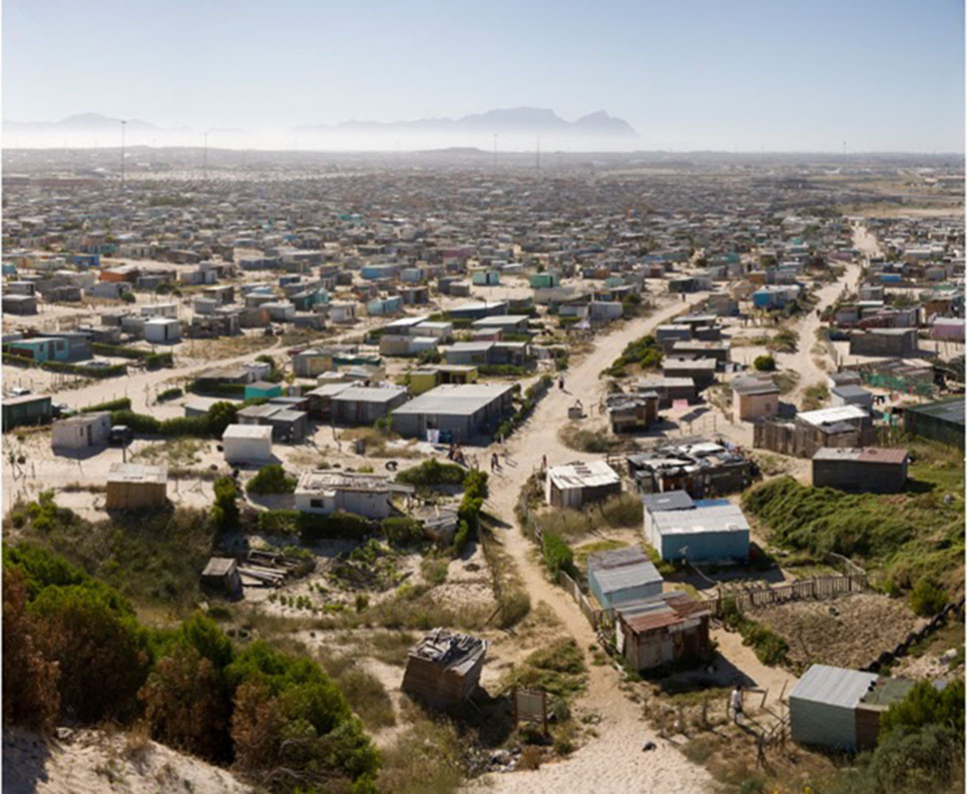
Informal neighborhoods in Cape Town are positioned such that residents are both more exposed to ecosystem disservices with shallow water tables and high volumes of mobile sand. A recent study also shows an additional layer of environmental injustice where these neighborhoods have less nature, less plant diversity, and less of the associated ecosystem services. Global change predictions will likely render these residents even more exposed when what little vegetation cover there is, is lost. (photography: Sean Wilson)

This study flags historical environmental injustices with black people marginalized to environmentally exposed spaces in the city, and then emergent ecologies that reflect the apartheid urban form and see the creation, and persistence, of environmental injustices (Fig. 7). While this study shows particular structural problems that might be attended to in some respects through city financial decision-making, the notion of access to nature and the plurality of values is of course complex. Tozer et al. (2020) point out that despite extensive literature it offers little by way of concrete advice for cities like Cape Town for effective redress. Tozer et al. (2020) go on to note that while research in this space takes a homogenous view on an ideal nature, it pays little attention to the social factors that should shape decision-making and the diversity of ways in which we make meaning of nature in our cities, and that this gap in research hinders progress in effectively addressing environmental injustices. In the case of Cape Town, while social and racial inequalities remain unresolved, in addition to allowing for greater social involvement in decision-making processes, any urban ecology work must likely engage competing or parallel social and development narratives around for example access to land, housing, livelihoods, and well-being.

### Balancing growth, ecology, and environmental justice in Bangalore

Bangalore, one of India’s largest and fastest growing cities, exemplifies many of the tensions between growth, equity and sustainability that characterizes most cities today—in particular, most fast-growing cities of the global South. The colonial footprint of the city’s past is visible in its contemporary ecology. The former British cantonment continues to be one of the most low-density parts of the city, with high green cover. These areas provide a stark contrast to the periphery, where expensive high-rise apartments, malls and corporate campuses share space with informal settlements that constitute sites of extreme poverty and deprivation. Bringing considerations of either ecology or environmental justice into discussions of planning becomes challenging – coupling them seems almost impossible.

Bangalore has a fairly high street and park tree diversity compared to many other cities (Nagendra and Gopal 2010, 2011). Many of these trees are imports, brought in by the British, or earlier rulers, for their large green canopies that provide shade in the tropics, and spectacular blossoms (Nagendra 2016). In the city’s parks, largely frequented by middle-class and wealthy residents, as many as four out of every five trees are exotic imports. Many of these ‘foreign’ trees can be considered naturalized, having been around for centuries. Much of the local insect and animal fauna has also co-evolved to use these trees for food, shelter and as nesting sites.

However, the dearth of native trees certainly affects the overall composition of biodiversity in Bangalore. It also impacts the city from an environmental justice point of view. Many low-income residents, especially new migrants to the city who are especially vulnerable because they lack social networks that provide additional safety nets, depend on parks, grassy open spaces, wetlands and lakes for foraging, harvesting close to a hundred different types of wild greens which they use as food, herbal medicine, and income supplementation. When parks and lakes are restored, the plant species selected are often non-native (Somesh et al. 2021). In addition, activities like foraging, grazing and subsistence fishing are actively discouraged, with restored ecosystems protected by fences, notices and guards (Nagendra 2016).

Informal settlements plant and carefully maintain a very high proportion of native species in tiny spaces, growing plants in broken buckets, battery cans, and any other bits of scrap they find at hand. Despite lacking open space, they nurture an impressive local biodiversity, and act as custodians of traditional ecological knowledge on the uses of native plants (Gopal et al. 2015). Yet, they are typically excluded from ‘consultative’ discussions on urban planning. Their expertise is dismissed by government planners, while their contributions to maintaining local biodiversity have rarely been documented. Wealthy resident groups often refer to them as the main contributors to the city’s degradation, dismissing their own role in influencing the growth policies of the city.

This not only exacerbates challenges of environmental injustice, but also further worsens the city’s ecology. As with many other global South cities, Bangalore’s infrastructure has not kept pace with urban expansion. Sewage frequently finds its way into lakes and wetlands, resulting in a high nitrogen and phosphorus load that leads to eutrophication. Grazing, which was once traditionally practiced in all lakes, is now banned in the city—especially in newly restored lakes. This impacts the income and livelihoods of traditional grazing communities and also reduces their capacity to remove biomass from the lake through ‘natural’ means, contributing to lake deterioration. Similarly, when fishers and foragers are banned from the lake, their experiential knowledge of local soil and hydrology, and indigenous ways of lake maintenance are also lost (Sen et al. 2021). In the few instances where lake associations have worked with fishers and residents of informal settlements, integrating their perspectives into lake management, the positive impacts on environmental justice and on ecology are visible (Nagendra 2016). Sadly, these are few and far between and have not been adopted as a city-wide approach toward urban planning—as they ought to be. Instead, restoration often follows an approach analogous to gentrification, excluding low-income residents with valuable traditional ecological knowledge, and further exacerbating their already precarious, marginal urban existence.

## Conclusion

We have used one of North America’s ongoing, long-term urban ecology research projects to illustrate the evolution of urban ecology toward greater attention to environmental justice scholarship and practice. We have also briefly shown the relevance of the shift in four other urban situations, spanning the Caribbean, Africa, and South Asia. We distill the following insights from the four cases discussed. This shift of urban ecology toward environmental justice is a response to three main things:

1. Increasingly deep engagement with communities, including those racially segregated or marginalized, alerted us to profound concern with racial and environmental justice. Among the networks required for this engagement were leaders and organizations that were dedicated to improvement of the social and environmental conditions in those oppressed communities. Because community engagement is required for successful urban long-term research, the project team necessarily became more proficient in dealing with environmental justice as a part of the urban social–ecological system.
2. Long-term transdisciplinary interaction across disciplines from social and biogeophysical sciences was required by the funding agency that originated the project, and this allowed the research to increasingly benefit from a plurality of scholarly perspectives. The team learned from each other through shared research projects and research locations in diverse communities. The resultant integration of data and concepts across the social and biophysical realms and exposure of new patterns and processes of environmental *IN*justice attracted attention in BES.
3. The surprising new patterns of distributional environmental injustice that we found required joint analysis of contemporary and historical data on social and biophysical interactions. Both a unified conception and a historical perspective reflecting dynamic heterogeneity proved necessary to explain both the expected and unexpected patterns we discovered on environmental justice in Baltimore.

A crucial new insight from BES is that the roots of environmental injustice in Baltimore and many other cities (Pulido 2000; Pellow 2004; Sze 2006; Benz 2019) are a highly resilient system of racism as a socio-political ideology. The theory of complex adaptive systems provided a theoretical and mechanistic basis for understanding the resilience of racism and exclusion. The history of environmental justice in Baltimore exposed a complex adaptive system that cycles through patterns of struggle and repression. Patterns of environmental inequity in Baltimore stimulate attempts by oppressed communities and progressive institutions to undo the drivers of inequity. In turn, those attempts at liberation are met with reaction by the elites in the city and federal establishment to stabilize segregation, exclusion, and extraction of wealth from the struggling communities. Thus, environmental inequity is a resilient system. This points to the reality that not all resilience is socially beneficial or ethical, and urban ecologists must take care to understand and act on this fact.

The resilience of oppression and injustice is common around the world. The case histories complimenting the Baltimore history show several kinds of oppression, and the efforts of communities and researchers to overcome the driving and resultant injustices in the Caribbean, Africa, and Asia. The paper on the “relational shift” in this special feature gives some additional examples relevant to this insight (Pickett et al. 2024).

Since Singh and other’s landmark publication in 2013, LTSER and urban LTER projects throughout the world continue to make substantial progress in multi-scale, transdisciplinary science: integrating social and biophysical sciences and humanities,;engaging diverse communities,;and connecting with decision-makers (Holzer et al. 2018; Grove and Pickett 2021; Holzer and Orenstein 2023). An emerging scientific and social frontier remains. Specifically, how do long-term, institutionalized and resilient forms of oppression and privilege come to be and adapt over time (Pickett et al. 2023). We expect that further transdisciplinary attention to environmental justice around the world will be fruitful. This is because the features revealed by the detailed history in Baltimore are far from unique. Racialized and other forms of segregation in urban systems are common (Nightingale 2012); long-term and historical research is growing globally (e.g., ILTER; Network—Global Coverage—ILTER—International long-term ecological research 2023); and integrated efforts by social and biophysical scientists are well established and growing. This area of focus may be crucial to the ability to create sustainable and resilient urban social–ecological systems. Through their existing foundations, LTSERs and urban LTERs are well positioned for this challenge.

## Footnotes

1 https://lternet.edu/network-organization/lter-a-history/.
